# Supplementary material for: Serratia marcescens Outbreak at a Correctional Facility: Environmental Sampling, Laboratory Analyses and Genomic Characterization to Assess Sources and Persistence
Source: Int J Environ Res Public Health. 2023 Sep 4;20(17):6709. doi: 10.3390/ijerph20176709 (PMC10487681; doi:10.3390/ijerph20176709)
Supplement: Supplementary file 1 [file ijerph-20-06709-s001.zip › ijerph-2510348 - Supplementary Material S2 (Figure S2).pdf]

Figure S2. Distance matrix (pairwise comparison). Value in intersection shows the number of SNPs distance between two isolates. Page 1 of 3.

More similar ⟶ More different

		1	2	3	4	5	6	7	8	9	10	11	12	13	14	15	16	17	18	19	20
Patient 5	1		0	0	0	0	0	0	0	0	0	0	0	0	0	0	0	0	0	0	0
Patient 1	2	132590		0	0	0	0	0	0	0	0	0	0	0	0	0	0	0	0	0	0
Patient 4	3	132594	6		0	0	0	0	0	0	0	0	0	0	0	0	0	0	0	0	0
Sample A-1	4	133680	25098	25102		0	0	0	0	0	0	0	0	0	0	0	0	0	0	0	0
Sample A-2	5	133680	25098	25102	2		0	0	0	0	0	0	0	0	0	0	0	0	0	0	0
Sample A-3	6	135197	25161	25165	25805	25805		0	0	0	0	0	0	0	0	0	0	0	0	0	0
Patient 8	7	178248	174564	174568	176080	176080	176141		0	0	0	0	0	0	0	0	0	0	0	0	0
Patient 17	8	134427	27171	27175	15989	15989	27266	176777		0	0	0	0	0	0	0	0	0	0	0	0
Patient 7-1	9	208544	193038	193042	194966	194966	195145	183026	195701		0	0	0	0	0	0	0	0	0	0	0
Patient 7-2	10	208544	193042	193046	194970	194970	195145	183028	195707	28		0	0	0	0	0	0	0	0	0	0
Patient 7-3	11	208540	193032	193036	194960	194960	195139	183026	195697	10	28		0	0	0	0	0	0	0	0	0
Patient 7-4	12	208546	193040	193044	194968	194968	195145	183026	195705	20	32	22		0	0	0	0	0	0	0	0
Sample U	13	50523	136421	136425	137511	137511	138706	180459	137980	211035	211029	211031	211037		0	0	0	0	0	0	0
Sample O	14	135885	30573	30577	19403	19403	31234	174529	21420	194997	195001	194991	194999	139728		0	0	0	0	0	0
Sample P	15	135886	30574	30578	19404	19404	31233	174530	21421	194998	195002	194992	195000	139729	1		0	0	0	0	0
Patient 2-1	16	132590	2	4	25098	25098	25161	174564	27171	193038	193042	193032	193040	136421	30573	30574		0	0	0	0
Sample V	17	46943	135317	135321	136363	136363	137750	178405	137466	207689	207685	207685	207683	44334	138496	138497	135317		0	0	0
Sample M-1	18	134662	26992	26996	13552	13552	27045	176830	17417	195668	195672	195662	195670	138403	20855	20856	26992	137063		0	0
Sample M-2	19	135125	26579	26583	27857	27857	26136	176967	29586	195231	195235	195225	195229	138940	32652	32651	26579	137782	29141		0
Sample D-1	20	134421	26905	26909	15649	15649	26890	176457	856	195351	195357	195347	195355	137996	21114	21113	26905	137172	17083	29254	
Sample D-2	21	134434	26720	26724	14700	14700	27057	176534	17341	195676	195680	195670	195678	138121	20363	20364	26720	137163	16584	29191	17045
Patient 2-2	22	132591	1	5	25099	25099	25160	174565	27172	193039	193043	193033	193041	136422	30574	30575	1	135318	26993	26578	26906
Patient 2-3	23	132589	1	5	25097	25097	25162	174563	27170	193037	193041	193031	193039	136420	30572	30573	1	135316	26991	26580	26904
Sample E-1	24	171834	170316	170320	171676	171676	172345	122992	172815	180206	180192	180202	180208	173795	171679	171680	170316	171967	172632	172581	172475
Sample E-2	25	171834	170314	170316	171676	171676	172339	122984	172813	180212	180200	180210	180216	173795	171677	171678	170312	171965	172632	172579	172473
Sample I-1	26	132589	1	5	25097	25097	25162	174563	27170	193037	193041	193031	193039	136420	30572	30573	1	135316	26991	26580	26904
Sample I-2	27	132590	0	6	25098	25098	25161	174564	27171	193038	193042	193032	193040	136421	30573	30574	2	135317	26992	26579	26905
Sample I-3	28	132589	1	7	25097	25097	25160	174563	27170	193037	193041	193031	193039	136420	30572	30573	3	135316	26991	26578	26904
Sample I-4	29	132589	1	5	25097	25097	25162	174563	27170	193037	193041	193031	193039	136420	30572	30573	1	135316	26991	26580	26904
Sample F	30	134986	24898	24902	25558	25558	845	176380	27171	194974	194974	194968	194974	138463	31311	31310	24898	137545	26740	25879	26731
Sample I-5	31	171839	170307	170309	171669	171669	172334	122983	172806	180205	180191	180201	180207	173796	171666	171667	170305	171970	172625	172572	172466
Sample N	32	135292	25252	25256	25910	25910	363	176228	27451	195232	195232	195226	195232	138787	31327	31326	25252	137845	27098	26245	27045
Sample Q	33	135931	30621	30623	19451	19451	31192	174575	21382	195045	195049	195039	195047	139774	56	55	30619	138544	20903	32694	21112
Patient 18	34	35157	133573	133577	134747	134747	136114	178841	135668	209153	209145	209151	209151	49342	136886	136887	133573	44930	135439	136188	135336
Patient 3-1	35	132589	1	5	25097	25097	25162	174563	27170	193037	193041	193031	193039	136420	30572	30573	1	135316	26991	26580	26904
Patient 3-2	36	132589	1	5	25097	25097	25162	174563	27170	193037	193041	193031	193039	136420	30572	30573	1	135316	26991	26580	26904
Sample H	37	15	132587	132591	133677	133677	135194	178243	134424	208543	208543	208539	208545	50518	135882	135883	132587	46938	134659	135122	134418
Sample B	38	14	132588	132592	133678	133678	135195	178242	134425	208544	208544	208540	208546	50519	135883	135884	132588	46939	134660	135123	134419
Sample C	39	132589	1	5	25097	25097	25162	174563	27170	193037	193041	193031	193039	136420	30572	30573	1	135316	26991	26580	26904
Patient 15	40	132590	2	6	25098	25098	25163	174564	27171	193038	193042	193032	193040	136421	30573	30574	2	135317	26992	26581	26905
Patient 6	41	209670	194334	194338	196302	196302	196219	186068	196589	52038	52036	52038	52036	210963	196361	196362	194334	208587	196706	196269	196273
Sample W	42	48332	135180	135184	136204	136204	137483	179526	136909	209348	209348	209346	209346	34217	138435	138436	135180	41997	137206	137711	136925
Sample Y	43	15	132595	132599	133687	133687	135204	178251	134434	208551	208551	208547	208553	50520	135892	135893	132595	46944	134669	135132	134428
Sample G	44	31199	132685	132689	133659	133659	135214	177907	134678	208299	208293	208297	208299	49566	135814	135815	132685	45126	134455	135172	134342
Sample R	45	135992	30670	30672	19662	19662	31199	174716	21617	195228	195232	195222	195230	139831	665	666	30668	138599	20906	32727	21363
Sample Z	46	209658	194320	194324	196288	196288	196211	186060	196579	52044	52044	52044	52044	210951	196347	196348	194320	208575	196702	196263	196263
Sample S	47	135962	30656	30660	19486	19486	31241	174604	21437	195082	195086	195076	195084	139805	131	130	30656	138581	20936	32729	21155
Sample T	48	135991	30669	30671	19661	19661	31196	174715	21616	195231	195235	195225	195233	139832	668	667	30667	138598	20905	32724	21360
Sample AB	49	134385	26673	26677	14657	14657	26928	176505	17340	195633	195637	195627	195635	138234	20320	20321	26673	137122	16547	28926	16998
Sample J	50	50243	136077	136081	137191	137191	138364	180133	137668	210709	210703	210705	210709	422	139384	139385	136077	44002	138083	138616	137688
Sample AA	51	50240	136076	136078	137188	137188	138361	180130	137665	210708	210706	210704	210708	425	139383	139384	136074	44003	138082	138613	137685
Sample K	52	50245	136073	136077	137185	137185	138360	180129	137662	210707	210701	210703	210707	418	139380	139381	136073	44004	138079	138612	137682
Sample L	53	135886	30574	30576	19406	19406	31229	174534	21423	195000	195004	194994	195002	139729	5	4	30572	138497	20858	32647	21115
Patient 9	54	132592	2	8	25100	25098	25161	174566	27171	193040	193044	193034	193042	136423	30575	30576	4	135319	26994	26579	26905
Patient 10	55	132592	4	6	25098	25098	25159	174566	27169	193040	193044	193034	193042	136423	30575	30576	2	135319	26994	26577	26903
Patient 11	56	132575	19	25	25083	25081	25144	174547	27154	193023	193027	193017	193025	136406	30558	30559	21	135302	26977	26562	26888
Patient 14	57	134681	27009	27013	13557	13557	27048	176843	17418	195683	195687	195677	195685	138420	20866	20867	27009	137082	39	29148	17084
Patient 13	58	132594	4	10	25100	25100	25161	174566	27171	193042	193046	193036	193044	136425	30577	30578	6	135321	26996	26579	26905
Patient 16	59	177829	175285	175287	176921	176921	176908	57559	177516	176007	175999	176007	176007	180404	175344	175345	175283	178120	177601	177164	177190
Patient 12	60	162802	161124	161128	162822	162822	162873	56346	163607	167736	167726	167734	167736	164849	161503	161504	161124	163071	163468	163221	163291

Figure S2 Continued. Page 2 of 3.

		21	22	23	24	25	26	27	28	29	30	31	32	33	34	35	36	37	38	39	40
Patient 5	1	0	0	0	0	0	0	0	0	0	0	0	0	0	0	0	0	0	0	0	0
Patient 1	2	0	0	0	0	0	0	0	0	0	0	0	0	0	0	0	0	0	0	0	0
Patient 4	3	0	0	0	0	0	0	0	0	0	0	0	0	0	0	0	0	0	0	0	0
Sample A-1	4	0	0	0	0	0	0	0	0	0	0	0	0	0	0	0	0	0	0	0	0
Sample A-2	5	0	0	0	0	0	0	0	0	0	0	0	0	0	0	0	0	0	0	0	0
Sample A-3	6	0	0	0	0	0	0	0	0	0	0	0	0	0	0	0	0	0	0	0	0
Patient 8	7	0	0	0	0	0	0	0	0	0	0	0	0	0	0	0	0	0	0	0	0
Patient 17	8	0	0	0	0	0	0	0	0	0	0	0	0	0	0	0	0	0	0	0	0
Patient 7-1	9	0	0	0	0	0	0	0	0	0	0	0	0	0	0	0	0	0	0	0	0
Patient 7-2	10	0	0	0	0	0	0	0	0	0	0	0	0	0	0	0	0	0	0	0	0
Patient 7-3	11	0	0	0	0	0	0	0	0	0	0	0	0	0	0	0	0	0	0	0	0
Patient 7-4	12	0	0	0	0	0	0	0	0	0	0	0	0	0	0	0	0	0	0	0	0
Sample U	13	0	0	0	0	0	0	0	0	0	0	0	0	0	0	0	0	0	0	0	0
Sample O	14	0	0	0	0	0	0	0	0	0	0	0	0	0	0	0	0	0	0	0	0
Sample P	15	0	0	0	0	0	0	0	0	0	0	0	0	0	0	0	0	0	0	0	0
Patient 2-1	16	0	0	0	0	0	0	0	0	0	0	0	0	0	0	0	0	0	0	0	0
Sample V	17	0	0	0	0	0	0	0	0	0	0	0	0	0	0	0	0	0	0	0	0
Sample M-1	18	0	0	0	0	0	0	0	0	0	0	0	0	0	0	0	0	0	0	0	0
Sample M-2	19	0	0	0	0	0	0	0	0	0	0	0	0	0	0	0	0	0	0	0	0
Sample D-1	20	0	0	0	0	0	0	0	0	0	0	0	0	0	0	0	0	0	0	0	0
Sample D-2	21		0	0	0	0	0	0	0	0	0	0	0	0	0	0	0	0	0	0	0
Patient 2-2	22	26721		0	0	0	0	0	0	0	0	0	0	0	0	0	0	0	0	0	0
Patient 2-3	23	26719	2		0	0	0	0	0	0	0	0	0	0	0	0	0	0	0	0	0
Sample E-1	24	172358	170317	170315		0	0	0	0	0	0	0	0	0	0	0	0	0	0	0	0
Sample E-2	25	172356	170313	170313	16		0	0	0	0	0	0	0	0	0	0	0	0	0	0	0
Sample I-1	26	26719	2	0	170315	170313		0	0	0	0	0	0	0	0	0	0	0	0	0	0
Sample I-2	27	26720	1	1	170316	170314	1		0	0	0	0	0	0	0	0	0	0	0	0	0
Sample I-3	28	26719	2	2	170315	170313	2	1		0	0	0	0	0	0	0	0	0	0	0	0
Sample I-4	29	26719	2	0	170315	170313	0	1	2		0	0	0	0	0	0	0	0	0	0	0
Sample F	30	26818	24897	24899	172128	172122	24899	24898	24897	24899		0	0	0	0	0	0	0	0	0	0
Sample I-5	31	172349	170306	170306	19	13	170306	170307	170306	170306	172117		0	0	0	0	0	0	0	0	0
Sample N	32	27182	25251	25253	172432	172426	25253	25252	25251	25253	858	172421		0	0	0	0	0	0	0	0
Sample Q	33	20411	30620	30620	171725	171721	30620	30621	30620	30620	31355	171710	31371		0	0	0	0	0	0	0
Patient 18	34	135391	133574	133572	172199	172201	133572	133573	133572	133572	135903	172206	136207	136934		0	0	0	0	0	0
Patient 3-1	35	26719	2	0	170315	170313	0	1	2	0	24899	170306	25253	30620	133572		0	0	0	0	0
Patient 3-2	36	26719	2	0	170315	170313	0	1	2	0	24899	170306	25253	30620	133572	0		0	0	0	0
Sample H	37	134431	132588	132586	171835	171835	132586	132587	132586	132586	134983	171840	135289	135928	35152	132586	132586		0	0	0
Sample B	38	134432	132589	132587	171840	171840	132587	132588	132587	132587	134984	171845	135290	135929	35155	132587	132587	11		0	0
Sample C	39	26719	2	0	170315	170313	0	1	2	0	24899	170306	25253	30620	133572	0	0	132586	132587		0
Patient 15	40	26720	3	1	170316	170314	1	2	3	1	24900	170307	25254	30621	133573	1	1	132587	132588	1	
Patient 6	41	196946	194335	194333	184816	184818	194333	194334	194333	194333	196036	184809	196290	196409	210553	194333	194333	209669	209670	194333	194334
Sample W	42	137046	135181	135179	172496	172496	135179	135180	135179	135179	137278	172499	137578	138481	47995	135179	135179	48329	48328	135179	135180
Sample Y	43	134441	132596	132594	171845	171845	132594	132595	132594	132594	134993	171850	135299	135938	35158	132594	132594	14	11	132594	132595
Sample G	44	134243	132686	132684	171435	171431	132684	132685	132684	132684	135003	171436	135309	135860	31098	132684	132684	31192	31193	132684	132685
Sample R	45	20268	30669	30669	171690	171686	30669	30670	30669	30669	31278	171675	31298	713	136987	30669	30669	135989	135990	30669	30670
Sample Z	46	196932	194321	194319	184814	184816	194319	194320	194319	194319	196028	184807	196282	196395	210541	194319	194319	209657	209658	194319	194320
Sample S	47	20442	30657	30655	171764	171762	30655	30656	30655	30655	31380	171751	31408	111	136971	30655	30655	135959	135960	30655	30656
Sample T	48	20267	30668	30668	171689	171685	30668	30669	30668	30668	31275	171678	31295	714	136986	30668	30668	135988	135989	30668	30669
Sample AB	49	531	26674	26672	172313	172311	26672	26673	26672	26672	26767	172304	27129	20286	135484	26672	26672	134382	134383	26672	26673
Sample J	50	137785	136078	136076	173467	173467	136076	136077	136076	136076	138121	173468	138445	139430	49022	136076	136076	50240	50243	136076	136077
Sample AA	51	137782	136075	136075	173466	173464	136075	136076	136075	136075	138118	173465	138442	139427	49027	136075	136075	50237	50238	136075	136076
Sample K	52	137779	136074	136072	173463	173463	136072	136073	136072	136072	138117	173464	138441	139426	49022	136072	136072	50240	50243	136072	136073
Sample L	53	20366	30573	30573	171680	171676	30573	30574	30573	30573	31306	171665	31322	57	136887	30573	30573	135883	135884	30573	30574
Patient 9	54	26722	3	3	170318	170316	3	2	3	3	24898	170309	25252	30623	133575	3	3	132589	132590	3	4
Patient 10	55	26722	3	3	170318	170314	3	4	5	3	24896	170307	25250	30621	133575	3	3	132589	132590	3	4
Patient 11	56	26705	20	20	170301	170299	20	19	20	20	24881	170292	25235	30606	133558	20	20	132572	132573	20	21
Patient 14	57	16593	27010	27008	172649	172649	27008	27009	27008	27008	26743	172642	27101	20914	135456	27008	27008	134678	134679	27008	27009
Patient 13	58	26724	5	5	170320	170318	5	4	5	5	24898	170311	25252	30625	133577	5	5	132591	132592	5	6
Patient 16	59	177365	175284	175284	120207	120199	175284	175285	175284	175284	176683	120198	176999	175388	178666	175284	175284	177824	177825	175284	175285
Patient 12	60	163424	161125	161123	102710	102704	161123	161124	161123	161123	162656	102705	162958	161551	163559	161123	161123	162797	162802	161123	161124

Figure S2 Continued. Page 3 of 3.

		41	42	43	44	45	46	47	48	49	50	51	52	53	54	55	56	57	58	59	60
Patient 5	1	0	0	0	0	0	0	0	0	0	0	0	0	0	0	0	0	0	0	0	0
Patient 1	2	0	0	0	0	0	0	0	0	0	0	0	0	0	0	0	0	0	0	0	0
Patient 4	3	0	0	0	0	0	0	0	0	0	0	0	0	0	0	0	0	0	0	0	0
Sample A-1	4	0	0	0	0	0	0	0	0	0	0	0	0	0	0	0	0	0	0	0	0
Sample A-2	5	0	0	0	0	0	0	0	0	0	0	0	0	0	0	0	0	0	0	0	0
Sample A-3	6	0	0	0	0	0	0	0	0	0	0	0	0	0	0	0	0	0	0	0	0
Patient 8	7	0	0	0	0	0	0	0	0	0	0	0	0	0	0	0	0	0	0	0	0
Patient 17	8	0	0	0	0	0	0	0	0	0	0	0	0	0	0	0	0	0	0	0	0
Patient 7-1	9	0	0	0	0	0	0	0	0	0	0	0	0	0	0	0	0	0	0	0	0
Patient 7-2	10	0	0	0	0	0	0	0	0	0	0	0	0	0	0	0	0	0	0	0	0
Patient 7-3	11	0	0	0	0	0	0	0	0	0	0	0	0	0	0	0	0	0	0	0	0
Patient 7-4	12	0	0	0	0	0	0	0	0	0	0	0	0	0	0	0	0	0	0	0	0
Sample U	13	0	0	0	0	0	0	0	0	0	0	0	0	0	0	0	0	0	0	0	0
Sample O	14	0	0	0	0	0	0	0	0	0	0	0	0	0	0	0	0	0	0	0	0
Sample P	15	0	0	0	0	0	0	0	0	0	0	0	0	0	0	0	0	0	0	0	0
Patient 2-1	16	0	0	0	0	0	0	0	0	0	0	0	0	0	0	0	0	0	0	0	0
Sample V	17	0	0	0	0	0	0	0	0	0	0	0	0	0	0	0	0	0	0	0	0
Sample M-1	18	0	0	0	0	0	0	0	0	0	0	0	0	0	0	0	0	0	0	0	0
Sample M-2	19	0	0	0	0	0	0	0	0	0	0	0	0	0	0	0	0	0	0	0	0
Sample D-1	20	0	0	0	0	0	0	0	0	0	0	0	0	0	0	0	0	0	0	0	0
Sample D-2	21	0	0	0	0	0	0	0	0	0	0	0	0	0	0	0	0	0	0	0	0
Patient 2-2	22	0	0	0	0	0	0	0	0	0	0	0	0	0	0	0	0	0	0	0	0
Patient 2-3	23	0	0	0	0	0	0	0	0	0	0	0	0	0	0	0	0	0	0	0	0
Sample E-1	24	0	0	0	0	0	0	0	0	0	0	0	0	0	0	0	0	0	0	0	0
Sample E-2	25	0	0	0	0	0	0	0	0	0	0	0	0	0	0	0	0	0	0	0	0
Sample I-1	26	0	0	0	0	0	0	0	0	0	0	0	0	0	0	0	0	0	0	0	0
Sample I-2	27	0	0	0	0	0	0	0	0	0	0	0	0	0	0	0	0	0	0	0	0
Sample I-3	28	0	0	0	0	0	0	0	0	0	0	0	0	0	0	0	0	0	0	0	0
Sample I-4	29	0	0	0	0	0	0	0	0	0	0	0	0	0	0	0	0	0	0	0	0
Sample F	30	0	0	0	0	0	0	0	0	0	0	0	0	0	0	0	0	0	0	0	0
Sample I-5	31	0	0	0	0	0	0	0	0	0	0	0	0	0	0	0	0	0	0	0	0
Sample N	32	0	0	0	0	0	0	0	0	0	0	0	0	0	0	0	0	0	0	0	0
Sample Q	33	0	0	0	0	0	0	0	0	0	0	0	0	0	0	0	0	0	0	0	0
Patient 18	34	0	0	0	0	0	0	0	0	0	0	0	0	0	0	0	0	0	0	0	0
Patient 3-1	35	0	0	0	0	0	0	0	0	0	0	0	0	0	0	0	0	0	0	0	0
Patient 3-2	36	0	0	0	0	0	0	0	0	0	0	0	0	0	0	0	0	0	0	0	0
Sample H	37	0	0	0	0	0	0	0	0	0	0	0	0	0	0	0	0	0	0	0	0
Sample B	38	0	0	0	0	0	0	0	0	0	0	0	0	0	0	0	0	0	0	0	0
Sample C	39	0	0	0	0	0	0	0	0	0	0	0	0	0	0	0	0	0	0	0	0
Patient 15	40	0	0	0	0	0	0	0	0	0	0	0	0	0	0	0	0	0	0	0	0
Patient 6	41		0	0	0	0	0	0	0	0	0	0	0	0	0	0	0	0	0	0	0
Sample W	42	209960		0	0	0	0	0	0	0	0	0	0	0	0	0	0	0	0	0	0
Sample Y	43	209677	48331		0	0	0	0	0	0	0	0	0	0	0	0	0	0	0	0	0
Sample G	44	209525	48233	31194		0	0	0	0	0	0	0	0	0	0	0	0	0	0	0	0
Sample R	45	196420	138544	135999	135913		0	0	0	0	0	0	0	0	0	0	0	0	0	0	0
Sample Z	46	34	209946	209665	209515	196414		0	0	0	0	0	0	0	0	0	0	0	0	0	0
Sample S	47	196446	138512	135969	135899	684	196432		0	0	0	0	0	0	0	0	0	0	0	0	0
Sample T	48	196423	138543	135998	135912	5	196417	685		0	0	0	0	0	0	0	0	0	0	0	0
Sample AB	49	196905	136991	134392	134336	20569	196891	20323	20566		0	0	0	0	0	0	0	0	0	0	0
Sample J	50	210635	33933	50240	49270	139487	210625	139461	139488	137898		0	0	0	0	0	0	0	0	0	0
Sample AA	51	210634	33930	50239	49275	139484	210622	139460	139485	137895	11		0	0	0	0	0	0	0	0	0
Sample K	52	210635	33931	50242	49272	139483	210623	139457	139484	137892	8	13		0	0	0	0	0	0	0	0
Sample L	53	196362	138436	135893	135815	668	196348	134	667	20321	139385	139382	139381		0	0	0	0	0	0	0
Patient 9	54	194336	135182	132597	132687	30672	194322	30658	30671	26675	136079	136078	136075	30576		0	0	0	0	0	0
Patient 10	55	194336	135182	132597	132687	30670	194322	30658	30669	26675	136079	136076	136075	30574	4		0	0	0	0	0
Patient 11	56	194323	135165	132580	132670	30655	194309	30641	30654	26658	136062	136061	136058	30559	19	21		0	0	0	0
Patient 14	57	196715	137221	134688	134474	20917	196711	20947	20916	16560	138100	138099	138096	20869	27011	27011	26994		0	0	0
Patient 13	58	194338	135184	132599	132689	30674	194324	30660	30673	26677	136081	136080	136077	30578	4	4	19	27013		0	0
Patient 16	59	179525	178707	177834	177886	175359	179533	175421	175356	177316	180086	180081	180084	175343	175287	175285	175270	177616	175289		0
Patient 12	60	170076	163748	162809	162641	161522	170070	161590	161521	163389	164509	164508	164505	161506	161126	161126	161109	163483	161128	53225	
