# Supplementary material for: Serratia marcescens Outbreak at a Correctional Facility: Environmental Sampling, Laboratory Analyses and Genomic Characterization to Assess Sources and Persistence
Source: Int J Environ Res Public Health. 2023 Sep 4;20(17):6709. doi: 10.3390/ijerph20176709 (PMC10487681; doi:10.3390/ijerph20176709)
Supplement: Supplementary file 1 [file ijerph-20-06709-s001.zip › ijerph-2510348 - Supplementary Material S3 (Table S1).pdf]

Table S1. Quality metrics and public repository information of the assembled whole genome sequences. Bolded samples are those selected for the PCA. NCBI ID was created by California Department of Public Health; therefore, is slightly different than this study (red text depicts a different ID). The NCBI Biosample Accession is linked to the raw reads and the assembled genome.

Sample ID (Sample ID-Isolate #; Source; Collection Date)	Phylogeny Group #	Assembly Quality								Public Repository	
		Contig #	N50	Coverage	Genome Length	GC%	N/ 100 kbp	BUSCO % complete	ORF Count	NCBI ID	Biosample Accession
**Patient 1: Blood; 2021-Jan-02**	1	58	5,301,542	169	5,394,984	59.0	122.5	96.8	5,133	PatientA	SAMN35676882
Patient 2-1: Blood; 2021-May-12	1	64	5,306,262	137	5,401,881	59.0	122.4	96.8	5,022	PatientB-1	SAMN35676883
Patient 2-2: Blood; 2021-May-12	1	57	5,309,218	219	5,391,866	59.0	118.8	96.8	5,006	PatientB-2	SAMN35676884
**Patient 2-3: Blood; 2021-May-12**	1	49	5,301,457	180	5,395,710	59.0	127.9	96.8	5,133	PatientB-3	SAMN35676885
**Patient 3-1: Epidural abscess ; 2021-Jul-06**	1	64	5,304,507	154	5,390,741	59.0	129.9	96.8	5,148	PatientD-1	SAMN35676886
Patient 3-2: Epidural abscess; 2021-Jul-06	1	64	5,304,994	178	5,399,198	59.0	128.0	96.8	5,022	PatientD-2	SAMN35676887
**Patient 4: Wound; 2021-Jan-24**	1	62	5,338,890	158	5,434,537	59.0	125.3	96.8	5,188	PatientE	SAMN35676888
**Patient 9: Blood; 2022-Jun-08**	1	46	5,300,599	216	5,390,180	59.0	122.4	96.8	5,127	PatientK	SAMN35676897
**Patient 10: Wound; 2022-Jul-11**	1	48	5,299,408	198	5,388,983	59.0	129.9	96.8	5,122	PatientL	SAMN35676898
**Patient 11: Blood; 2022-Aug-03**	1	46	5,298,675	184	5,388,797	59.0	129.9	96.8	5,130	PatientM	SAMN35676899
Patient 13: Abscess Tissue; 2022-Oct-13	1	62	5,278,662	102	5,373,929	59.1	121.1	96.8	4,990	PatientO	SAMN35676901
Patient 15: Blood; 2021-Aug-10	1	59	5,306,933	158	5,401,047	59.0	122.4	96.8	5,019	PatientR	SAMN35676904
**Sample C: Nasacort w/ Methamphetamine; 2021-Jul-06**	1	67	5,302,635	182	5,388,975	59.0	129.9	96.8	5,143	SampleC	SAMN35676912
**Sample I-1: Surrendered Needles; 2021-May-17**	1	54	5,304,295	195	5,375,050	59.0	115.4	96.8	5,113	SampleI-1	SAMN35676920
Sample I-2: Surrendered Needles; 2021-May-17	1	55	5,302,865	195	5,393,292	59.0	107.7	96.8	5,002	SampleI-2	SAMN35676921
**Sample I-3: Surrendered Needles; 2021-May-17**	1	62	5,299,365	159	5,368,825	59.0	121.1	96.8	5,116	SampleI-3	SAMN35676922
Sample I-4: Surrendered Needles; 2021-May-17	1	56	5,304,592	176	5,394,463	59.0	115.1	96.8	5,005	SampleI-4	SAMN35676923
**Sample A-3: CB64 Dilution Machine; 2021-Mar-05**	2	83	5,123,078	150	5,435,345	58.8	167.4	96.8	5,194	SampleA-1	SAMN35676906
**Sample F: Scrubbie 3; 2021-May-17**	2	68	5,165,856	173	5,370,501	58.9	141.5	96.8	5,152	SampleF	SAMN35676917
Sample M-2: Mop bucket; 2021-May-07	0	67	5,331,327	122	5,405,278	58.9	161.2	96.8	5,075	SampleM-1	SAMN35676928
Sample N: CB64 in Bottle; 2021-May-17	2	99	5,185,830	167	5,419,545	58.8	131.3	96.8	5,056	SampleN	SAMN35676930
Patient 14: Urine catheter; 2022-Oct-09	4	97	5,262,059	133	5,530,557	58.6	150.4	96.8	5,171	PatientP	SAMN35676902
**Sample A-1: CB64 Dilution Machine; 2021-Mar-05**	0	54	5,309,929	182	5,478,639	59.3	140.7	96.8	5,207	SampleA-2	SAMN35676907
Sample A-2: CB64 Dilution Machine; 2021-Mar-05	0	49	5,324,940	106	5,474,114	59.6	115.2	96.8	5,112	SampleA-3	SAMN35676908
Sample AB: Break Out from Trash Can; 2021-Aug-4	5	65	5,295,535	95	5,456,494	59.1	165.2	96.8	5,064	SampleAB	SAMN35676910
**Sample D-2: Scrubbie; 2021-May-07**	5	69	5,526,998	172	5,247,456	59.0	153.8	96.8	5,258	SampleD-1	SAMN35676913
Sample M-1: Mop bucket; 2021-May-07	4	99	5,285,922	139	5,528,670	58.7	132.3	96.8	5,213	SampleM-2	SAMN35676929
**Patient 17: Joint Fluid; 2021-Mar-12**	3	89	5,397,984	164	5,636,381	59.0	118.9	96.8	5,459	PatientJ	SAMN35676896
Sample O: CB64 in Bottle; 2021-Apr-21	6	69	5,258,061	175	5,328,953	59.3	94.0	96.8	4,985	SampleO	SAMN35676931
**Sample L: Diluted CB64; 2021-Aug-04**	6	68	5,209,298	141	5,307,802	59.3	111.2	96.8	5,055	SampleL	SAMN35676927
Sample D-1: Scrubbie; 2021-May-07	5	101	5,336,904	155	5,530,767	59.2	135.9	96.8	5,224	SampleD-2	SAMN35676914
Sample P: CB64 in Coffee Container; 2021-Apr-21	6	78	5,251,314	177	5,327,907	58.7	97.8	96.8	4,992	SampleP	SAMN35676932
Sample Q: Empty Gatorade Bottle; 2021-May-17	6	75	5,210,729	202	5,343,117	59.2	97.5	96.8	4,984	SampleQ	SAMN35676933
Sample R: Diluted Break Out from Trash Can 1; 2021-Aug-4	6	88	5,138,946	199	5,384,944	58.9	195.3	96.8	5,015	SampleR	SAMN35676934
Sample S: Front Door Floor, D3-125; 2021-Sep-01	6	74	5,238,087	161	5,358,166	59.1	102.8	96.8	4,995	SampleS	SAMN35676935
Sample T: Front Door Floor; 2021-Aug-4	6	87	5,139,978	155	5,380,664	58.9	193.6	96.8	5,010	SampleT	SAMN35676936
Sample AA: Bottle used as Urinal; 2021-Aug-13	7	38	5,149,531	175	5,328,097	59.7	43.2	96.0	4,868	SampleAA	SAMN35676909
**Sample J: Detergent; 2021-Aug-13**	7	18	5,148,442	197	5,326,547	59.7	33.8	96.0	4,856	SampleJ	SAMN35676925
**Sample K: Cleaner; 2021-Aug-13**	7	16	5,148,532	143	5,315,593	59.7	37.6	96.0	4,853	SampleK	SAMN35676926
Sample U: Drinking Water in Bottle; 2021-Apr-21	7	37	5,443,673	174	5,545,244	59.6	37.9	96.0	5,112	SampleU	SAMN35676937
Sample V: Break Out; 2021-May-07	0	98	5,140,277	115	5,195,109	60.1	56.0	95.2	4,812	SampleV	SAMN35676938
Sample W: Floor around Door; 2021-Aug-04	0	35	5,316,523	174	5,416,490	59.8	51.7	95.2	5,028	SampleW	SAMN35676939
**Patient 5: Blood; 2021-Jan-11**	8	36	5,187,190	148	5,240,895	59.9	49.7	95.2	4,869	PatientF	SAMN35676889
Patient 18: Blood; 2021-May-19	0	36	5,128,270	199	5,141,614	60.0	44.8	94.3	4,713	PatientS	SAMN35676905
**Sample B: Coffee from Cup; 2021-Jul-06**	8	35	5,186,963	159	5,229,629	59.9	44.0	95.2	4,866	SampleB	SAMN35676911
**Sample G: Shower Floor; 2021-Aug-04**	0	30	5,049,776	170	5,054,870	60.1	29.7	94.3	4,666	SampleG	SAMN35676918
**Sample H: Sterile Saline Hand Rinsate; 2021-Jul-08**	8	35	5,186,972	178	5,229,638	59.9	45.9	95.2	4,866	SampleH	SAMN35676919
Sample Y: Floor Around Door; 2021-Aug-04	8	39	5,166,058	198	5,243,685	59.9	45.8	95.2	4,862	SampleY	SAMN35676940
**Patient 6: Wound; 2021-Aug-28**	10	33	5,031,850	250	5,038,040	59.6	43.7	96.0	4,728	PatientG	SAMN35676890
Patient 7-1: Urine Catheter; 2021-Apr-09	9	23	4,997,111	172	5,007,736	59.7	28.0	96.0	4,623	PatientH-1	SAMN35676891
**Patient 7-2: Blood; 2021-Apr-09**	9	24	4,996,931	174	5,004,684	59.7	30.0	96.0	4,687	PatientH-2	SAMN35676892
Patient 7-3: Back Wound; 2021-Apr-13	9	23	4,997,213	169	5,007,838	59.7	30.0	96.0	4,620	PatientH-3	SAMN35676893
Patient 7-4: Back Wound; 2021-Apr-13	9	24	4,996,894	191	5,007,694	59.7	28.0	96.0	4,622	PatientH-4	SAMN35676894
Sample E-1: Scrubbie; 2021-May-17	11	26	4,944,991	201	4,955,211	59.8	30.3	96.0	4,582	SampleE-1	SAMN35676915
Sample Z: Bottle To Store Water; 2021-Sept-1	10	36	5,031,036	213	5,044,026	59.6	49.6	96.0	4,656	SampleZ	SAMN35676941
**Patient 8: Urine Catheter; 2021-Mar-11**	0	38	5,271,589	187	5,287,127	59.3	35.9	96.0	5,056	PatientI	SAMN35676895
**Patient 12: Knee; 2022-April-08**	0	16	5,087,127	145	5,098,696	59.7	41.2	96.0	4,759	PatientN	SAMN35676900
Patient 16: Sputum; 2022-Oct-12	0	17	5,143,969	156	5,152,647	59.7	42.7	96.0	4,785	PatientQ	SAMN35676903
**Sample E-2: Scrubbie; 2021-May-17**	11	23	4,945,474	230	4,951,361	59.8	34.3	96.0	4,586	SampleE-2	SAMN35676916
Sample I-5: Surrendered Needles; 2021-May-17	11	26	4,944,943	195	4,955,162	59.8	30.3	96.0	4,582	SampleI-5	SAMN35676924
