# Supplementary material for: Serratia marcescens Outbreak at a Correctional Facility: Environmental Sampling, Laboratory Analyses and Genomic Characterization to Assess Sources and Persistence
Source: Int J Environ Res Public Health. 2023 Sep 4;20(17):6709. doi: 10.3390/ijerph20176709 (PMC10487681; doi:10.3390/ijerph20176709)
Supplement: Supplementary file 1 [file ijerph-20-06709-s001.zip › ijerph-2510348 - Supplementary Material S4 (Tables S2 and S3).pdf]

Table S2. Raw and standardized counts of eight categories of virulence genes.

Sample ID	Virulence		Betain/Proline		Biofilm		Fimbria		Flagel		Hemolysin		Siderophore		Toxins		Total
(Sample ID-Isolate#; Source; Collection Date)	Count	Standard	Count	Standard	Count	Standard	Count	Standard	Count	Standard	Count	Standard	Count	Standard	Count	Standard	
Patient 1: Blood; 2021-Jan-02	8	0.17	13	-0.33	5	0.41	33	-0.62	40	0.19	18	0.44	10	-0.63	50	0.99	177
Patient 2-3: Blood; 2021-May-12	8	0.17	13	-0.33	5	0.41	33	-0.62	40	0.19	18	0.44	10	-0.63	49	0.82	176
Patient 3-1: Epidural abscess ; 2021-Jul-06	8	0.17	13	-0.33	5	0.41	33	-0.62	40	0.19	18	0.44	10	-0.63	49	0.82	176
Patient 4: Wound; 2021-Jan-24	8	0.17	13	-0.33	5	0.41	33	-0.62	40	0.19	18	0.44	10	-0.63	50	0.99	177
Patient 9: Blood; 2022-Jun-08	8	0.17	13	-0.33	5	0.41	33	-0.62	40	0.19	18	0.44	10	-0.63	51	1.15	178
Patient 10: Wound; 2022-Jul-11	8	0.17	13	-0.33	5	0.41	33	-0.62	40	0.19	18	0.44	10	-0.63	50	0.99	177
Patient 11: Blood; 2022-Aug-03	8	0.17	13	-0.33	5	0.41	31	-0.95	40	0.19	18	0.44	10	-0.63	49	0.82	174
Sample C: Nasacort w/ Methamphetamine; 2021-Jul-06	9	0.51	13	-0.33	5	0.41	33	-0.62	40	0.19	18	0.44	10	-0.63	49	0.82	177
Sample I-1: Surrendered Needles; 2021-May-17	8	0.17	13	-0.33	5	0.41	33	-0.62	40	0.19	18	0.44	10	-0.63	50	0.99	177
Sample I-3: Surrendered Needles; 2021-May-17	8	0.17	13	-0.33	5	0.41	33	-0.62	40	0.19	18	0.44	10	-0.63	49	0.82	176
Sample A-3: CB64 Dilution Machine; 2021-Mar-05	8	0.17	12	-2.13	5	0.41	27	-1.60	40	0.19	18	0.44	9	-0.99	51	1.15	170
Sample F: Scrubbie 3; 2021-May-17	13	1.84	13	-0.33	5	0.41	32	-0.79	40	0.19	18	0.44	9	-0.99	49	0.82	179
Sample A-1: CB64 Dilution Machine; 2021-Mar-05	8	0.17	15	3.26	5	0.41	41	0.68	40	0.19	16	-0.82	10	-0.63	46	0.32	181
Sample D-2: Scrubbie; 2021-May-07	18	3.52	14	1.46	5	0.41	35	-0.30	40	0.19	20	1.70	10	-0.63	49	0.82	191
Patient 17: Joint Fluid; 2021-Mar-12	8	0.17	14	1.46	5	0.41	37	0.03	40	0.19	19	1.07	10	-0.63	45	0.15	178
Sample L: Diluted CB64; 2021-Aug-04	9	0.51	13	-0.33	5	0.41	31	-0.95	40	0.19	18	0.44	11	-0.28	40	-0.69	167
Sample G: Shower Floor; 2021-Aug-04	4	-1.16	13	-0.33	5	0.41	43	1.01	40	0.19	17	-0.19	15	1.14	35	-1.52	172
Sample K: Cleaner; 2021-Aug-13	5	-0.83	13	-0.33	5	0.41	42	0.85	40	0.19	18	0.44	18	2.21	38	-1.02	179
Sample J: Detergent; 2021-Aug-13	5	-0.83	13	-0.33	5	0.41	42	0.85	40	0.19	18	0.44	18	2.21	38	-1.02	179
Patient 5: Blood; 2021-Jan-11	4	-1.16	13	-0.33	5	0.41	41	0.68	39	-2.41	17	-0.19	15	1.14	38	-1.02	172
Sample B: Coffee from Cup; 2021-Jul-06	4	-1.16	13	-0.33	5	0.41	41	0.68	39	-2.41	17	-0.19	15	1.14	38	-1.02	172
Sample H: Sterile Saline Hand Rinsate; 2021-Jul-08	4	-1.16	13	-0.33	5	0.41	41	0.68	39	-2.41	17	-0.19	15	1.14	38	-1.02	172
Patient 6: Wound; 2021-Aug-28	6	-0.50	13	-0.33	4	-2.35	41	0.68	40	0.19	13	-2.70	15	1.14	39	-0.86	171
Patient 7-2: Blood; 2021-Apr-09	7	-0.16	13	-0.33	4	-2.35	54	2.80	40	0.19	14	-2.07	9	-0.99	41	-0.52	182
Patient 8: Urine Catheter; 2021-Mar-11	9	0.51	14	1.46	5	0.41	45	1.34	41	2.79	18	0.44	15	1.14	38	-1.02	185
Patient 12: Knee; 2022-April-08	4	-1.16	14	1.46	4	-2.35	44	1.17	40	0.19	15	-1.45	11	-0.28	40	-0.69	172
Sample E-2: Scrubbie; 2021-May-17	5	-0.83	13	-0.33	4	-2.35	29	-1.28	40	0.19	14	-2.07	13	0.43	32	-2.03	150

Table S3. Loadings of each virulence-related gene category for each principal component (PC) from principal components analysis and the percent variation explained (shaded in gray) by each PC.

	PC 1	PC 2	PC 3	PC 4	PC 5	PC 6	PC 7	PC 8
Virulence Reg	0.414	0.299	0.119	-0.014	-0.321	0.754	0.122	-0.197
Proline/Betaine	-0.023	0.485	0.552	-0.422	-0.316	-0.405	0.105	0.085
Hemolysin	0.419	-0.287	0.401	0.090	0.060	0.078	-0.372	0.652
Biofilm	0.339	-0.434	0.436	-0.011	0.153	-0.193	-0.003	-0.668
Siderophore	-0.374	-0.317	0.384	0.366	-0.198	0.137	0.625	0.173
Fimbria	-0.381	0.194	0.363	-0.199	0.703	0.378	-0.088	-0.027
Toxins	0.477	0.062	-0.189	-0.204	0.439	-0.124	0.660	0.216
Flagellar	0.157	0.513	0.125	0.773	0.207	-0.219	-0.041	-0.074
**Percent Variation Explained**	43.111	20.206	16.048	9.094	4.800	4.429	1.404	0.907
