# Supplementary material for: Serratia marcescens Outbreak at a Correctional Facility: Environmental Sampling, Laboratory Analyses and Genomic Characterization to Assess Sources and Persistence
Source: Int J Environ Res Public Health. 2023 Sep 4;20(17):6709. doi: 10.3390/ijerph20176709 (PMC10487681; doi:10.3390/ijerph20176709)
Supplement: Supplementary file 1 [file ijerph-20-06709-s001.zip › ijerph-2510348 - Supplementary Material S5 (Table S4).pdf]

Table S4. Counts of anti-microbial resistance (AMR) genes identified in the assembled genomes.

Sample IDs	Total	BETA-LACTAM				AMINOGLYCOSIDE	AMINOGLYCOSIDE/ QUINOLONE	BLEOMYCIN	FOSFOMYCIN	MACROLIDE	PHENICOL	QUINOLONE	STREPTOTHRICIN	SULFONAMIDE	TETRACYCLINE
		A	B	C	D										
Patient 1: Blood; 2021-Jan-02	4			1		1			1						1
Patient 2-1: Blood; 2021-May-12	4			1		1			1						1
Patient 2-2: Blood; 2021-May-12	4			1		1			1						1
Patient 2-3: Blood; 2021-May-12	4			1		1			1						1
Patient 3-1: Epidural abscess ; 2021-Jul-06	4			1		1			1						1
Patient 3-2: Epidural abscess; 2021-Jul-06	4			1		1			1						1
Patient 4: Wound; 2021-Jan-24	4			1		1			1						1
Patient 5: Blood; 2021-Jan-11	3			1		1			1						
Patient 6: Wound; 2021-Aug-28	4			1		1			1						1
Patient 7-1: Urine Catheter; 2021-Apr-09	4			1		1			1						1
Patient 7-2: Blood; 2021-Apr-09	4			1		1			1						1
Patient 7-3: Back Wound; 2021-Apr-13	4			1		1			1						1
Patient 7-4: Back Wound; 2021-Apr-13	4			1		1			1						1
Patient 8: Urine Catheter; 2021-Mar-11	4			1		1			1						1
Patient 9: Blood; 2022-Jun-08	4			1		1			1						1
Patient 10: Wound; 2022-Jul-11	4			1		1			1						1
Patient 11: Blood; 2022-Aug-03	4			1		1			1						1
Patient 12: Knee; 2022-April-08	4			1		1			1						1
Patient 13: Abscess Tissue; 2022-Oct-13	4			1		1			1						1
Patient 14: Urine catheter; 2022-Oct-09	4			1		1			1						1
Patient 15: Blood; 2021-Aug-10	4			1		1			1						1
Patient 16: Sputum; 2022-Oct-12	4			1		1			1						1
Patient 17: Joint Fluid; 2021-Mar-12	4			1		1			1						1
Patient 18: Blood; 2021-May-19	3			1		1			1						
Sample A-1: CB64 Dilution Machine; 2021-Mar-05	6			1		3			1						1
Sample A-2: CB64 Dilution Machine; 2021-Mar-05	6			1		3			1						1
Sample A-3: CB64 Dilution Machine; 2021-Mar-05	3			1					1						1
Sample B: Coffee from Cup; 2021-Jul-06	3			1		1			1						
Sample C: Nasacort w/ Methamphetamine; 2021-Jul-06	4			1		1			1						1
Sample D-1: Scrubbie; 2021-May-07	4			1		1			1						1
Sample D-2: Scrubbie; 2021-May-07	8			1		2			1			2	1		1
Sample E-1: Scrubbie; 2021-May-17	4			1		1			1						1
Sample E-2: Scrubbie; 2021-May-17	4			1		1			1						1
Sample F: Scrubbie 3; 2021-May-17	3			1					1						1
Sample G: Shower Floor; 2021-Aug-04	3			1		1			1						
Sample H: Sterile Saline Hand Rinsate; 2021-Jul-08	3			1		1			1						
Sample I-1: Surrendered Needles; 2021-May-17	4			1		1			1						1
Sample I-2: Surrendered Needles; 2021-May-17	4			1		1			1						1
Sample I-3: Surrendered Needles; 2021-May-17	4			1		1			1						1
Sample I-4: Surrendered Needles; 2021-May-17	4			1		1			1						1
Sample I-5: Surrendered Needles; 2021-May-17	4			1		1			1						1
Sample J: Detergent; 2021-Aug-13	3			1		1			1						
Sample K: Cleaner; 2021-Aug-13	3			1		1			1						
Sample L: Diluted CB64; 2021-Aug-04	4			1		1			1						1
Sample M-1: Mop bucket; 2021-May-07	4			1		1			1						1
Sample M-2: Mop bucket; 2021-May-07	4			1		1			1						1
Sample N: CB64 in Bottle; 2021-May-17	3			1					1						1
Sample O: CB64 in Bottle; 2021-Apr-21	4			1		1			1						1
Sample P: CB64 in Coffee Container; 2021-Apr-21	4			1		1			1						1
Sample Q: Empty Gatorade Bottle; 2021-May-17	4			1		1			1						1
Sample R: Diluted Break Out from Trash Can 1; 2021-Aug-4	3			1		1			1						
Sample S: Front Door Floor, D3-125; 2021-Sep-01	4			1		1			1						1
Sample T: Front Door Floor; 2021-Aug-4	3			1		1			1						
Sample U: Drinking Water in Bottle; 2021-Apr-21	3			1		1			1						
Sample V: Break Out; 2021-May-07	3			1		1			1						
Sample W: Floor around Door; 2021-Aug-04	3			1		1			1						
Sample Y: Floor Around Door; 2021-Aug-04	3			1		1			1						
Sample Z: Bottle To Store Water; 2021-Sept-1	4			1		1			1						1
Sample AA: Bottle used as Urinal; 2021-Aug-13	3			1		1			1						
Sample AB: Break Out from Trash Can; 2021-Aug-4	4			1		1			1						1
GCF_003031645.1 (Reference)	11	3		1	1	4			1						1
Serratia_liquefaciens_GCF_008364325	4			1					1			1			1
Clincial_Germany_GCF_900029885.1	4			1		1			1						1
Clinical_Japan_GCF_000828775	8		1	2		2			1					2	
Clinical_Mexico_GCF_001294565	4	1		1		1			1						
Clinical_Romania_GCF_002810285	15	1	1	1	1	5	1	1	1		1			2	
Clinical_Unknown_GCF_002947235	3			1		1			1						
Clinical_Unknown_GCF_002996885	4	1		1		1			1						
Clinical_USA_GCF_000783915.2	3			1		1			1						
Clinical_USA_GCF_001022215	13	3		1	1	3			1	2				1	1
Clinical_USA_GCF_002220515	4			1		1			1						1
Clinical_USA_GCF_002220535	4			1		1			1						1
Clinical_USA_GCF_002220615	4			1		1			1						1
Envi_China_GCF_001417865	4			1		1			1						1
Envi_Sweden_GCF_000513215	4			1		1			1						1
Envi_Taiwan_GCF_000336425	4			1		1			1						1
