# Supplementary figures and images for: Serratia marcescens Outbreak at a Correctional Facility: Environmental Sampling, Laboratory Analyses and Genomic Characterization to Assess Sources and Persistence
Source: Int J Environ Res Public Health. 2023 Sep 4;20(17):6709. doi: 10.3390/ijerph20176709 (PMC10487681; doi:10.3390/ijerph20176709)

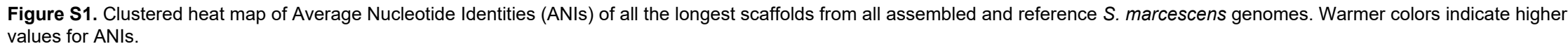

Supplement: Supplementary file 1 [file ijerph-20-06709-s001.zip › ijerph-2510348 - Supplementary Material S1 (Figure S1).pdf]
